# Hydroxyapatite-Mediated Mechanical Modulation of GelMA Hydrogels Influences Osteogenic Differentiation of 3D Spheroids

**DOI:** 10.3390/gels12010092

**Published:** 2026-01-20

**Authors:** Narantungalag Amarbayasgalan, Ji Hyeon Kim, Won-Gun Koh, Karthika Muthuramalingam, Hyun Jong Lee

**Affiliations:** 1 Department of Chemical and Biological Engineering, Gachon University, 1342 Seongnam-daero, Seongnam-si 13120, Gyeonggi-do, Republic of Korea; 2 Department of Chemical and Biomolecular Engineering, Yonsei University, 50 Yonsei-ro, Seodaemun-gu, Seoul 03722, Republic of Korea

**Keywords:** GelMA hydrogel, hydroxyapatite, mechanotransduction, osteogenic differentiation, spheroid culture

## Abstract

Substrate stiffness critically regulates osteogenic differentiation, yet systematic identification of optimal mechanical conditions in three-dimensional culture remains limited. This study investigated how hydroxyapatite (HAp)-mediated mechanical modulation of gelatin methacryloyl (GelMA) hydrogels influences osteogenic differentiation of encapsulated SAOS-2 spheroids. GelMA hydrogels with HAp at 5, 10, and 15 μg/mL were characterized for mechanical properties and used to encapsulate pre-formed spheroids under osteogenic conditions. GelMA+HAp5 achieved the highest compressive modulus, while higher HAp concentrations reduced crosslinking efficiency. All formulations maintained comparable viability and metabolic activity. Notably, GelMA+HAp10 produced the highest alkaline phosphatase activity at Days 7 and 14, despite lower stiffness than GelMA+HAp5, demonstrating a non-linear relationship between substrate mechanics and osteogenic response. These results establish that optimizing rather than maximizing mechanical properties represents a more effective scaffold design strategy for bone tissue engineering.

## 1. Introduction

Bone fractures are among the most common injuries worldwide, with large bone defects and critical-sized fractures presenting particularly challenging clinical scenarios [1,2,3]. While small fractures typically heal spontaneously, extensive bone loss resulting from trauma, tumor resection, or infection frequently leads to delayed union or nonunion [4,5,6]. Current therapeutic strategies, including autologous bone grafting, are constrained by limited donor tissue availability, harvest site morbidity, and insufficient biological integration [5,7]. These limitations underscore the need for engineered bone substitutes that provide both mechanical support and biological cues to promote bone regeneration.

The success of bone regeneration strategies depends critically on recreating the mechanobiological environment of native bone tissue. Cells actively probe and respond to the mechanical properties of their surrounding matrix through mechanotransduction pathways [8,9]. Substrate stiffness serves as a fundamental regulator of osteogenic differentiation, with cells preferentially differentiating toward osteogenic lineages on substrates with bone-like mechanical properties [10,11]. Importantly, osteogenic differentiation exhibits a biphasic response: substrates that are too compliant fail to provide sufficient mechanical stimulation, while overly rigid substrates can inhibit differentiation efficiency [11]. This “optimal stiffness window” concept has profound implications for scaffold design, yet systematic approaches to achieve precise mechanical tuning in three-dimensional hydrogel systems remain limited.

Despite the recognized importance of mechanical cues, most bone tissue engineering approaches have focused primarily on biochemical properties. Hydroxyapatite (HAp), the mineral component of natural bone, has been extensively incorporated into biomaterials for its osteoconductive properties, but typically as a structural filler without systematic consideration of its role as a mechanical modulator [12,13]. Similarly, gelatin methacryloyl (GelMA), a photocrosslinkable hydrogel with cell-adhesive RGD motifs, has gained attention for bone tissue engineering, yet often lacks the mechanical strength to mimic native bone tissue and provide optimal mechanotransduction cues [14,15]. The challenge lies in developing a systematic strategy to modulate hydrogel mechanical properties and to identify the optimal stiffness range for osteogenesis in three-dimensional culture systems.

The three-dimensional architecture of cell culture systems fundamentally influences how cells perceive and respond to mechanical cues. Traditional two-dimensional cultures impose artificial constraints and distort mechanotransduction signaling [16,17]. Three-dimensional spheroid cultures enable cells to establish physiological cell–cell contacts and experience mechanical forces distributed throughout a three-dimensional volume, more accurately recapitulating the mechanical microenvironment of bone tissue [18,19]. Encapsulation of pre-formed spheroids within hydrogel matrices combines three-dimensional cellular organization with controlled extracellular mechanical environment, creating a powerful platform for studying mechanotransduction in osteogenesis [20]. SAOS-2 cells, a well-characterized osteoblastic cell line with high alkaline phosphatase activity, form robust spheroids and serve as an established model for investigating osteogenic differentiation [21,22].

We propose a strategy that positions HAp as a “mechanical tuning agent” to systematically modulate the stiffness of GelMA hydrogels. By incorporating HAp at defined concentrations, the compressive modulus can be incrementally adjusted to span the optimal stiffness range for osteogenic differentiation. This approach leverages HAp’s dual functionality: its osteoconductive properties complement its mechanical reinforcement effects, potentially creating synergistic benefits [23,24]. The combination of GelMA’s cell-adhesive properties with HAp’s mechanical and biochemical contributions offers a rational framework for creating biomimetic scaffolds, while the dose-dependent design enables establishing quantitative relationships between mechanical properties and biological outcomes.

In this study, we systematically investigate how HAp-mediated mechanical reinforcement of GelMA hydrogels influences the osteogenic differentiation of encapsulated SAOS-2 spheroids. We prepared GelMA hydrogels with varying HAp concentrations (5, 10, and 15 μg/mL) and characterized their structural and mechanical properties. Pre-formed SAOS-2 spheroids were encapsulated and cultured under osteogenic conditions, with evaluation of viability, metabolic activity, alkaline phosphatase activity, and matrix mineralization. By correlating mechanical properties with biological responses across a range of HAp concentrations, this work aims to identify whether an optimal stiffness window exists for osteogenic differentiation in three-dimensional spheroid cultures and to establish design principles for mechanically tuned hydrogel scaffolds.

## 2. Results and Discussion

### 2.1. Design and Fabrication of HAp-Reinforced GelMA Hydrogels

To systematically investigate the influence of mechanical properties on osteogenic differentiation in three-dimensional culture, we developed a hydrogel-based platform that enables precise control of substrate stiffness through hydroxyapatite (HAp) incorporation. Gelatin methacryloyl (GelMA) was selected as the base hydrogel due to its photocrosslinkable nature, inherent cell-adhesive properties derived from collagen, and established biocompatibility [25,26]. HAp was incorporated at three concentrations (5, 10, and 15 μg/mL) to achieve dose-dependent mechanical reinforcement while maintaining the hydrogel’s capacity for cell encapsulation.

A key design consideration in this study was the use of pre-formed spheroids rather than single-cell suspensions for hydrogel encapsulation. Conventional approaches disperse individual cells within hydrogel precursor solutions, requiring cells to establish new contacts and organize into functional units after encapsulation. This process can take several days and may be influenced by hydrogel properties in ways that confound mechanotransduction studies. In contrast, our approach encapsulates pre-formed SAOS-2 spheroids that already possess established cell–cell junctions, nascent extracellular matrix, and three-dimensional tissue-like organization [27]. This strategy offers several advantages: spheroids enter the hydrogel environment as functional multicellular units capable of immediate collective mechanosensing; the preserved cellular organization enables cells to experience mechanical cues through both cell–matrix and cell–cell interactions; and the spheroid architecture more closely mimics the cellular arrangement found in developing bone tissue. Furthermore, pre-formed spheroids provide consistent initial conditions across experimental groups, ensuring that observed differences in osteogenic responses can be attributed to the mechanical environment rather than variations in cell aggregation or organization processes.

**Figure 1:**
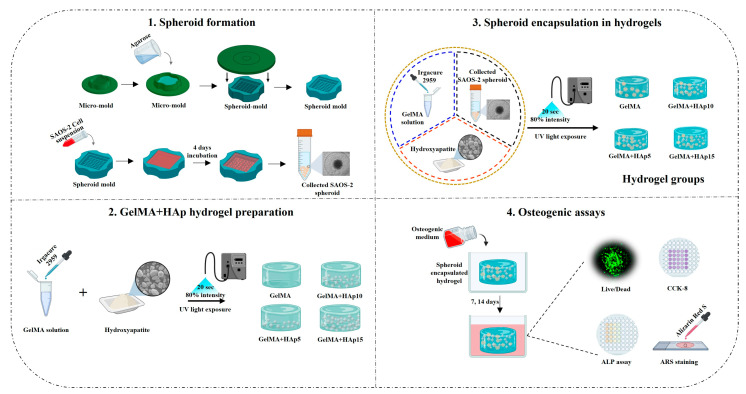
Experimental design for studying mechanotransduction-driven osteogenesis in HAp-reinforced hydrogels. The workflow consists of (**1**) SAOS-2 spheroid formation in agarose micro-molds, (**2**) GelMA-HAp hydrogel preparation with varying HAp concentrations, (**3**) spheroid encapsulation via UV photocrosslinking, and (**4**) evaluation of cell viability and osteogenic differentiation.

This spheroid encapsulation approach combines the benefits of defined cellular organization with a controllable extracellular mechanical environment, creating an integrated system for studying mechanotransduction in osteogenesis (Figure 1). The experimental workflow proceeded through spheroid formation in agarose microwells, encapsulation within photocrosslinked GelMA-HAp hydrogels, and subsequent culture under osteogenic conditions for up to 14 days, with systematic evaluation of mechanical properties, cell viability, and osteogenic differentiation markers.

### 2.2. Structural Characterization of HAp-Incorporated Hydrogels

**Figure 2:**
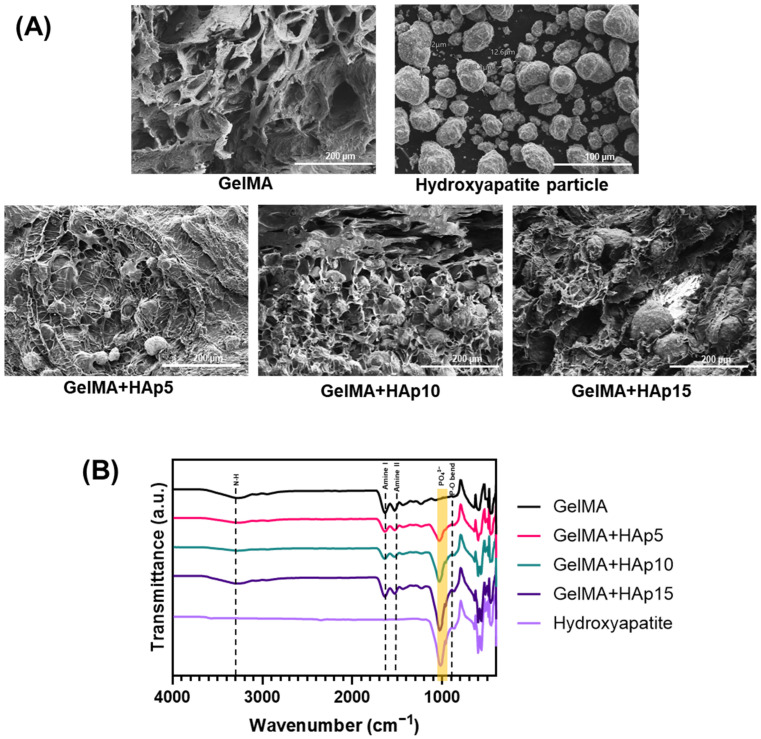
Structural characterization of hydroxyapatite-incorporated GelMA hydrogels. (**A**) Scanning electron microscopy (SEM) images of lyophilized hydrogel samples showing the internal porous architecture. Scale bars: 200 μm (hydrogel images), 100 μm (HAp particle image). (**B**) Fourier-transform infrared (FTIR) spectra of GelMA and GelMA+HAp hydrogels.

The successful incorporation and distribution of HAp within the GelMA matrix were first confirmed through scanning electron microscopy (SEM) analysis of lyophilized hydrogel samples (Figure 2A). Pure GelMA hydrogels exhibited a smooth, homogeneous porous architecture with interconnected pore networks characteristic of photocrosslinked gelatin-based hydrogels. The porous structure, resulting from ice crystal formation during lyophilization followed by sublimation, provides pathways for nutrient diffusion and waste removal—critical factors for maintaining cell viability in three-dimensional culture systems [28]. Upon HAp incorporation, notable morphological changes were observed in a dose-dependent manner. At 5 μg/mL HAp, discrete particles appeared distributed throughout the polymer matrix, while at 10 and 15 μg/mL, particle density increased substantially with maintained uniform distribution (Figure 2A). High-magnification imaging of HAp particles revealed their spherical morphology with diameters predominantly in the 1–5 μm range, consistent with the commercially obtained HAp specification. Importantly, even at the highest concentration tested (15 μg/mL), HAp particles remained well-dispersed without significant agglomeration, attributable to the sonication step employed during hydrogel preparation [29]. The preservation of porous architecture in HAp-incorporated samples, albeit with slight densification, suggests that the composite hydrogels maintain adequate permeability for molecular transport while gaining structural reinforcement from the mineral phase.

The chemical composition of the hydrogels was further analyzed using Fourier-transform infrared spectroscopy (FTIR) to confirm HAp incorporation (Figure 2B). Pure GelMA displayed characteristic amide bands at approximately 1650 cm^−1^ (amide I, C=O stretching) and 1550 cm^−1^ (amide II, N-H bending), along with amide A band near 3300 cm^−1^ (N-H stretching), typical of gelatin-derived materials [25,30]. HAp-containing hydrogels exhibited additional prominent peaks in the 1000–1100 cm^−1^ region, corresponding to phosphate (PO_4_^3−^) stretching vibrations, and a peak near 960 cm^−1^ attributed to P-O bending modes, both diagnostic of the hydroxyapatite crystalline structure [31]. The intensity of these phosphate-related peaks increased progressively with HAp concentration, providing quantitative evidence of dose-dependent incorporation. The position and overall shape of the amide bands remained largely unchanged, indicating that HAp incorporation did not induce major chemical modification or disruption of the primary GelMA polymer network. Notably, minor intensity variations and subtle band broadening in the amide and phosphate regions at higher HAp contents suggest weak physical interactions between GelMA functional groups and the HAp surface, without evidence of new covalent bond formation. These findings confirm successful integration of HAp into the GelMA matrix while preserving the fundamental hydrogel architecture.

### 2.3. Mechanical Property Modulation Through HAp Incorporation

**Figure 3:**
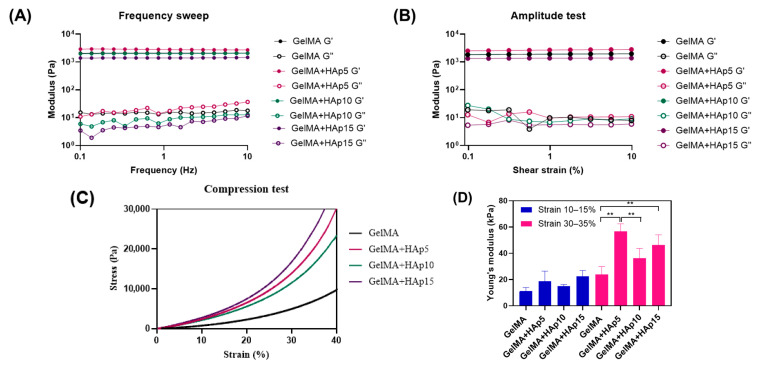
Mechanical property modulation of GelMA hydrogels through dose-dependent HAp incorporation. (**A**) Frequency sweep rheological analysis showing storage modulus (G′, solid symbols) and loss modulus (G″, open symbols) as functions of oscillatory frequency (0.1–10 Hz) for pure GelMA and GelMA+HAp composites. Data presented as mean ± SD (*n* = 3). (**B**) Amplitude sweep measurements showing storage modulus (G′) and loss modulus (G″) as functions of shear strain (0.1–10%) for all hydrogel formulations. Data presented as mean ± SD (*n* = 3). (**C**) Representative stress–strain curves from uniaxial compression testing (0–40% strain) showing non-linear elastic behavior with clear separation between formulations. (**D**) Quantification of compressive modulus calculated from the linear region (10–15% strain, 30–35% strain) of stress–strain curves. ** *p* < 0.01 compared to GelMA control. Error bars represent SD (*n* = 3).

Substrate stiffness represents a critical determinant of cellular mechanotransduction and osteogenic fate decisions [32]. Rheological analysis was employed to assess the viscoelastic behavior of the hydrogels across a physiologically relevant frequency range (Figure 3A). Frequency sweep measurements revealed that the storage modulus (G′, representing elastic behavior) consistently exceeded the loss modulus (G″, representing viscous behavior) across all tested frequencies for all formulations, confirming the formation of stable, gel-like networks [33]. G′ values remained relatively constant across the frequency range tested (0.1–10 Hz), indicating frequency-independent behavior characteristic of well-crosslinked polymer networks.

The relationship between HAp concentration and storage modulus exhibited a non-monotonic pattern. At 1 Hz, the storage modulus increased from approximately 2 kPa for pure GelMA to ~2.85 kPa for GelMA+HAp5, indicating effective reinforcement of the hydrogel network at low HAp concentration. This enhancement can be attributed to HAp particles acting as additional physical crosslinking points within the GelMA matrix. However, further increases in HAp content to 10 and 15 μg/mL resulted in progressively lower G′ values (~2.0 kPa and ~1.4 kPa, respectively). This reduction may indicate that higher HAp loading influences photocrosslinking efficiency, potentially through increased light scattering and steric hindrance and/or weak interfacial interactions that locally restrict polymer chain mobility and methacryloyl group accessibility, leading to a reduced effective crosslinking density. Amplitude sweep measurements (Figure 3B) confirmed that all formulations maintained stable network structures within the tested strain range (0.1–10%), demonstrating that the hydrogels can withstand physiological deformation levels without structural disruption [34].

Compressive mechanical properties were assessed through uniaxial compression testing, a loading mode particularly relevant to bone tissue (Figure 3C). The stress–strain curves displayed non-linear elastic behavior typical of hydrogel materials, with increasing resistance to compression at higher strain levels. Quantification of the compressive modulus from the linear regions of the stress–strain curves (10–15% and 30–35% strain) revealed a distinct pattern (Figure 3D). Pure GelMA exhibited the lowest compressive modulus, which increased substantially with incorporation of 5 μg/mL HAp. This formulation achieved the highest compressive modulus among all groups, reflecting the combined effects of optimal crosslinking density (as indicated by the highest G′ in rheological measurements) and the reinforcing contribution of HAp particles. At 10 μg/mL HAp, the compressive modulus decreased compared to HAp5, consistent with the reduced crosslinking density observed in rheological analysis. At 15 μg/mL HAp, the modulus increased relative to HAp10; although the crosslinking density was further reduced, the higher HAp particle content provided sufficient mechanical reinforcement to partially compensate for the weaker polymer network. This suggests that at high HAp concentrations, the direct mechanical contribution of rigid HAp particles becomes the dominant factor influencing compressive properties.

The mechanical reinforcement achieved through HAp incorporation can be attributed to synergistic mechanisms. HAp particles function as rigid fillers within the compliant GelMA matrix, creating a composite structure where applied loads are transferred from the polymer phase to the stiffer inorganic phase [29]. The uniform distribution of HAp particles throughout the hydrogel network, as confirmed by SEM analysis (Figure 2A), ensures efficient load transfer and prevents stress concentration that could arise from particle agglomeration [35]. Additionally, interactions between HAp particle surfaces and GelMA polymer chains—potentially involving hydrogen bonding between phosphate groups and gelatin’s amino acid residues—may contribute to network stabilization [35]. However, these reinforcing effects must be balanced against the potential interference with photocrosslinking at higher HAp concentrations.

The stiffness range achieved in this study lies within regimes reported to regulate osteogenic differentiation in three-dimensional (3D) hydrogel systems with elastic or bulk stiffness values of approximately 6–30 kPa [36,37]. Early work demonstrated that mesenchymal stem cells preferentially undergo osteogenic differentiation on two-dimensional, highly crosslinked substrates with elastic moduli of approximately 25–40 kPa [32]; however, these values are not directly transferable to viscoelastic 3D matrices. Subsequent work has demonstrated that both excessively compliant and rigid substrates can impair osteogenic differentiation [38]. Soft substrates may fail to provide sufficient mechanical resistance for robust integrin clustering and focal adhesion maturation [39], while overly rigid substrates may restrict cellular processes such as cytoskeletal remodeling [40]. Our systematic modulation of GelMA stiffness through controlled HAp incorporation positions the experimental groups across a mechanobiologically relevant range, enabling investigation of whether an optimal stiffness window exists for osteogenic differentiation in three-dimensional spheroid cultures.

The photocrosslinkable nature of GelMA provides spatial and temporal control over gelation, enabling potential applications in injectable or 3D-printed bone tissue engineering strategies where in situ curing is advantageous [26]. The comprehensive mechanical characterization presented here provides the foundation for interpreting subsequent biological responses within a mechanotransduction framework.

### 2.4. Biocompatibility and Cell Viability in HAp-GelMA Hydrogels

**Figure 4:**
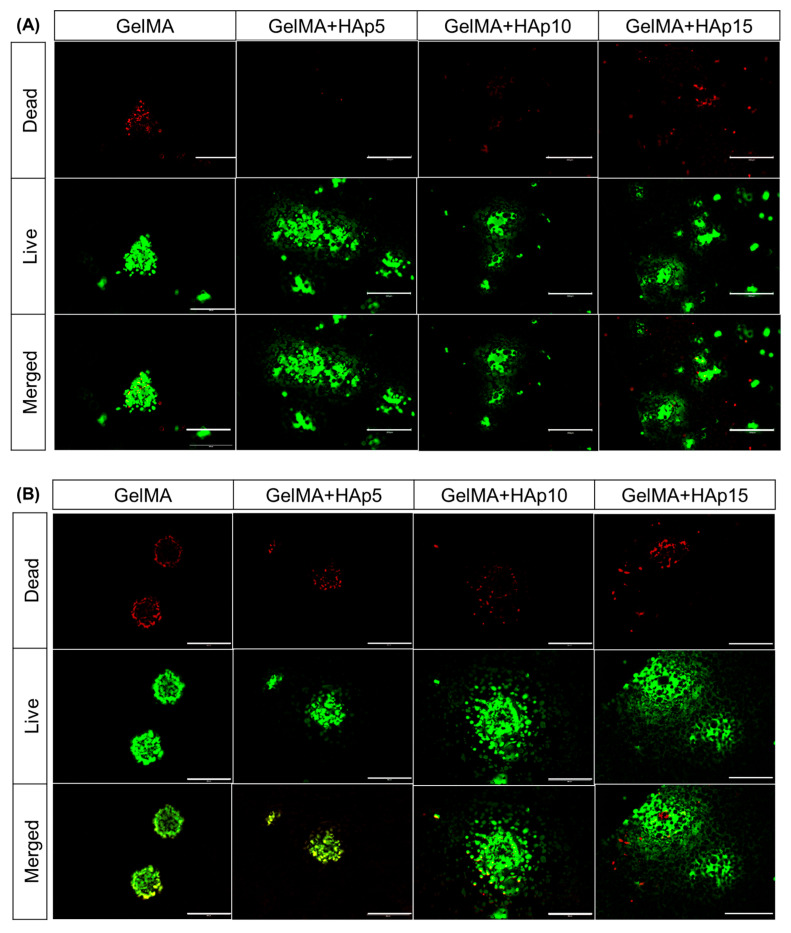

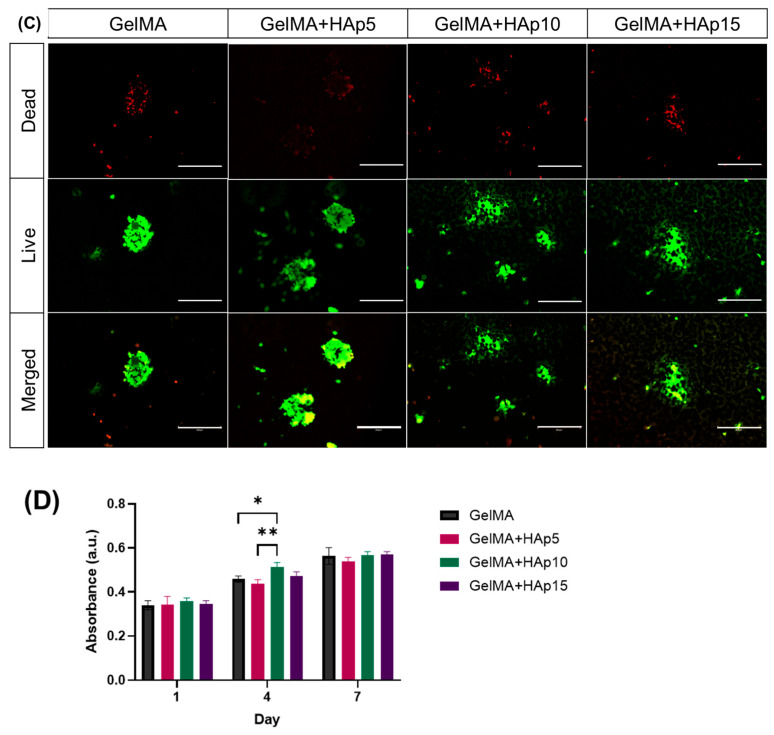
Biocompatibility and metabolic activity of SAOS-2 spheroids encapsulated in HAp-GelMA hydrogels. Live/Dead fluorescence staining of encapsulated spheroids at Day 1 (**A**), Day 4 (**B**), and Day 7 (**C**) post-encapsulation. Scale bars: 300 μm. (**D**) CCK-8 metabolic activity assay quantification at Days 1, 4, and 7. Data presented as mean ± SD (*n* = 3). * *p* < 0.05 and ** *p* < 0.01.

The cytocompatibility of HAp-GelMA composite hydrogels and their capacity to support three-dimensional spheroid culture were assessed through Live/Dead fluorescence staining and metabolic activity measurements. Live/Dead staining at Days 1, 4, and 7 post-encapsulation revealed predominantly green fluorescence (live cells) across all hydrogel formulations, with red fluorescence (dead cells) also consistently present throughout the culture period (Figure 4A–C). At Day 1, all groups displayed strong green fluorescence with intact spheroid morphology, confirming that neither the encapsulation process nor the UV photocrosslinking step induced acute cytotoxicity [41]. The presence of dead cells at similar levels across all formulations, including pure GelMA, reflects the inherent characteristics of three-dimensional spheroid culture rather than material-induced toxicity. In spheroid cultures, cells in the core region naturally experience limited oxygen and nutrient diffusion, leading to a baseline level of cell death that is independent of the surrounding matrix composition [28]. This pattern persisted through Days 4 and 7, with spheroids maintaining their compact three-dimensional architecture across all groups. The comparable distribution of live and dead cells among GelMA, GelMA+HAp5, GelMA+HAp10, and GelMA+HAp15 indicates that HAp incorporation at the tested concentrations does not introduce additional cytotoxicity beyond that inherent to three-dimensional spheroid culture.

Metabolic activity was quantified using the CCK-8 assay at Days 1, 4, and 7 (Figure 4D). All experimental groups showed gradual increases in absorbance over the culture period, indicating maintained cellular metabolic function within the encapsulated spheroids. At each time point, absorbance values were comparable across all formulations, with no substantial differences observed between pure GelMA and HAp-containing groups. This result suggests that HAp incorporation, despite altering the mechanical properties of the hydrogels, does not significantly affect cellular metabolic activity within the tested concentration range. The similar metabolic profiles across mechanically distinct environments indicate that the viability and basic cellular functions of encapsulated spheroids are maintained regardless of HAp content.

These findings demonstrate that all HAp-GelMA formulations provide a cytocompatible environment for three-dimensional spheroid culture. The mechanical property differences achieved through HAp incorporation (as characterized in Section 2.3) do not translate into differences in cell viability or metabolic activity under the conditions tested. This outcome is consistent with the known biocompatibility of both GelMA and HAp: GelMA provides cell-adhesive RGD sequences that support cell attachment and survival [14,15], while HAp, as the primary mineral component of natural bone, releases calcium and phosphate ions at physiological levels that do not impair cellular function [42,43,44]. The preservation of spheroid viability across all mechanical formulations establishes that subsequent differences in osteogenic differentiation markers can be attributed to mechanobiological effects rather than cytotoxicity-related artifacts.

### 2.5. Osteogenic Differentiation in Mechanically Modulated Hydrogels

**Figure 5:**
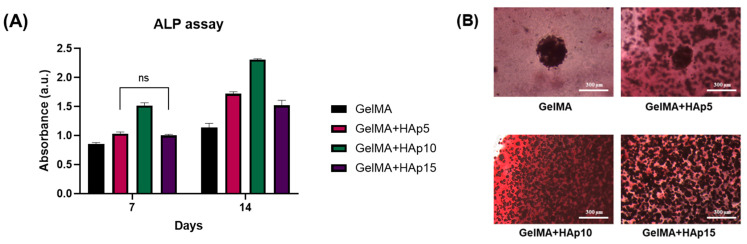
Dose-dependent osteogenic differentiation of SAOS-2 spheroids reveals an optimal mechanical window. (**A**) Alkaline phosphatase (ALP) activity measured at Days 7 and 14 post-encapsulation. Data presented as mean ± SD (*n* = 3). Statistical comparisons were performed within each time point. ns, not significant; all other comparisons *p* < 0.01. (**B**) Representative images of Alizarin Red S (ARS) staining at Day 14, visualizing calcium deposition and matrix mineralization. Images are presented for qualitative assessment only. Scale bars: 300 μm.

The central hypothesis of this study was that systematic modulation of hydrogel mechanical properties influences osteogenic differentiation of encapsulated spheroids, even when cell viability and metabolic activity remain unaffected. Alkaline phosphatase (ALP), a key early-stage osteogenic marker that catalyzes the hydrolysis of phosphate esters to provide inorganic phosphate for mineralization, was assessed at Days 7 and 14 (Figure 5A). We note that ALP is an early marker of osteogenic differentiation and does not alone indicate complete osteogenesis; therefore, its activity was interpreted alongside qualitative ARS staining to provide complementary insights into early-stage osteogenic responses. At Day 7, GelMA+HAp10 exhibited the highest ALP activity among all groups, while GelMA+HAp5 and GelMA+HAp15 showed similar intermediate levels with no significant difference between them. By Day 14, ALP activity increased across all groups, and the pattern became more pronounced: GelMA+HAp10 maintained the highest activity, with all HAp-containing groups significantly exceeding pure GelMA. The temporal progression from Day 7 to Day 14 confirms active osteogenic processes within the encapsulated spheroids.

These ALP results reveal an important finding when considered alongside the mechanical characterization data. Although GelMA+HAp5 exhibited the highest compressive modulus among all formulations (Figure 3D), it did not produce the strongest osteogenic response. Instead, GelMA+HAp10 consistently showed the highest ALP activity despite having a lower compressive modulus than HAp5. This dissociation between mechanical stiffness and osteogenic outcome suggests that the relationship between substrate mechanics and osteogenic differentiation is not simply linear. The enhanced osteogenic response in HAp10 may reflect an optimal balance between mechanical cues and other factors. HAp incorporation simultaneously modulates hydrogel mechanical properties and releases calcium and phosphate ions that serve as biochemical signals for osteogenesis [42,43,44]. Both of these effects may exhibit optimal ranges: just as excessively stiff or compliant matrices can impair differentiation, excessive ion concentrations from higher HAp content could potentially inhibit rather than promote osteogenic responses. The reduced ALP activity in HAp15 compared to HAp10, despite higher mineral content, may result from suboptimal mechanical conditions associated with the lowest crosslinking density, excessive ion release, or a combination of both factors. In the current experimental design, the relative contributions of mechanical and biochemical effects cannot be completely decoupled; however, the non-monotonic relationship between HAp concentration and osteogenic outcomes suggests that the system operates within an optimal regime where both factors may act in concert.

To assess late-stage mineralization, Alizarin Red S (ARS) staining was performed on Day 14 samples to visualize calcium deposition (Figure 5B). Pure GelMA exhibited minimal red staining, while HAp-containing groups showed increased staining intensity. GelMA+HAp10 appeared to demonstrate more intense staining compared to other groups, consistent with the ALP activity pattern. In the GelMA+HAp10 and GelMA+HAp15 groups, the optical opacity of dispersed HAp particles, combined with intense ARS staining, reduced image contrast and made spheroid boundaries difficult to distinguish, consistent with the reduced spheroid visibility observed in fluorescence imaging of these groups (Figure 4A–C). Given the three-dimensional spheroid architecture and the presence of pre-existing mineral within the matrix, mineral staining was therefore interpreted qualitatively and used to support trends observed in ALP activity rather than as a standalone quantitative measure.

The finding that osteogenic differentiation responds differently to mechanical environment than cell viability and metabolic activity is consistent with the established understanding of mechanotransduction in osteogenesis. Cells can maintain basic survival and metabolic functions across a relatively broad range of mechanical conditions, but lineage-specific differentiation programs are more sensitive to mechanical cues [45,46]. The mechanotransduction pathways that regulate osteogenic gene expression—involving integrin-mediated adhesion, focal adhesion kinase signaling, and downstream transcription factor activation—respond to substrate mechanics in ways that influence differentiation efficiency without necessarily affecting cell survival [8,46,47]. In our system, all formulations supported comparable viability (Section 2.4), but the osteogenic response showed clear dependence on HAp concentration, with GelMA+HAp10 providing conditions most favorable for ALP expression.

The three-dimensional spheroid culture system employed in this study enables collective mechanosensing that may differ from single-cell responses to substrate mechanics. Within spheroids, cells experience mechanical cues through both cell–matrix interactions at the spheroid periphery and cell–cell interactions throughout the spheroid structure [48,49]. This integrated mechanical environment may explain why the optimal formulation for osteogenesis (HAp10) does not correspond to the formulation with highest bulk stiffness (HAp5). The mechanical signals experienced by cells in three-dimensional spheroid culture reflect a complex integration of matrix properties, cellular contractility, and intercellular force transmission that cannot be fully predicted from bulk mechanical measurements alone.

The identification of GelMA+HAp10 as the formulation producing the strongest early osteogenic response provides a basis for scaffold optimization in bone tissue engineering applications. Rather than maximizing HAp content or mechanical stiffness, these results suggest that intermediate formulations may achieve superior biological outcomes. Future studies incorporating additional osteogenic markers and extended culture periods would further clarify the optimal conditions for bone tissue engineering scaffolds.

### 2.6. Study Limitations

While this study demonstrates that HAp incorporation modulates both the mechanical environment and osteogenic responses in 3D GelMA hydrogels, several considerations merit discussion. The effects of HAp arise from a combination of matrix stiffening and bioactive ion-mediated signaling, which were intentionally examined together but not independently decoupled in the present design. Notably, both mechanical properties and ion release may exhibit optimal ranges, and the observed peak response at HAp10 could reflect optimization of either or both factors. Bulk mechanical measurements capture baseline elastic properties of the matrix but may not fully represent microscale mechanical cues at the spheroid–matrix interface that contribute to cell mechanosensing. Interpretation of mineralization outcomes in HAp-containing, 3D spheroid-based hydrogels also warrants caution. Unlike HAp-free scaffold systems where ARS selectively binds to cell-deposited calcium, ARS in HAp-containing matrices binds to both pre-existing scaffold-associated calcium and newly formed mineral, generating background signals that vary with HAp concentration across experimental groups. Additionally, the three-dimensional spheroid architecture limits planar imaging depth resolution, and optical opacity from dispersed HAp particles further constrains visualization of cell-mediated deposition, a constraint inherent to mineral-containing scaffold systems rather than specific to the present experimental design; accordingly, mineral staining was used as qualitative support alongside ALP activity, which served as the primary quantitative indicator of early osteogenic differentiation. Future studies employing HAp-free mechanical modulators, direct ion release measurements, or molecular-level osteogenic analyses will enable clearer decoupling of mechanical and biochemical effects and more definitive assessment of mineralization.

## 3. Conclusions

This study systematically investigated the influence of hydroxyapatite (HAp)-mediated mechanical modulation of GelMA hydrogels on the osteogenic differentiation of encapsulated SAOS-2 spheroids. HAp incorporation produced distinct mechanical profiles, with GelMA+HAp5 exhibiting the highest compressive modulus due to optimal crosslinking density, while higher HAp concentrations reduced crosslinking efficiency. All formulations maintained comparable cell viability and metabolic activity, indicating that the tested stiffness range does not adversely affect basic cellular functions. The key finding was that GelMA+HAp10 produced the highest alkaline phosphatase activity despite not having the highest mechanical stiffness, demonstrating a non-linear relationship between substrate mechanics and osteogenic differentiation. Alizarin Red S staining provided qualitative support for enhanced mineralization, though the three-dimensional spheroid architecture and HAp-induced optical opacity limited quantitative assessment. These findings establish that optimizing the mechanical environment, rather than maximizing stiffness or mineral content, represents a more effective strategy for bone tissue engineering scaffold design.

## 4. Materials and Methods

### 4.1. Materials

Gelatin methacryloyl (GelMA) was purchased from CLECELL (Seongnam-si, Gyeonggi-do, Republic of Korea). Hydroxyapatite (HAp) and the photoinitiator Irgacure 2959 were obtained from Sigma-Aldrich (St. Louis, MO, USA). SAOS-2 human osteoblast-like cells were obtained from the Korean Cell Line Bank (Seoul, Republic of Korea). Cell culture reagents including Dulbecco’s Modified Eagle Medium (DMEM), fetal bovine serum (FBS), 100× penicillin/streptomycin (P/S), and trypsin-EDTA were purchased from WelGene (Gyeongsan, Republic of Korea). Spheroid-forming agarose molds (Z764019-6EA) were obtained from Sigma-Aldrich Korea. The LIVE/DEAD™ Viability/Cytotoxicity Kit and Cell Counting Kit-8 (CCK-8) were purchased from Thermo Fisher Scientific (Waltham, MA, USA). The alkaline phosphatase (ALP) assay kit and Alizarin Red S (ARS) were acquired from Sigma-Aldrich Korea. Unless otherwise specified, all chemicals and solvents were used as received from the manufacturers.

### 4.2. Hydrogel Fabrication and Characterization (SEM, FTIR)

Gelatin methacryloyl (GelMA) hydrogels were prepared at 10% (*w*/*v*) by dissolving GelMA in PBS containing photoinitiator Irgacure 2959 (0.5% *w*/*v*). Hydroxyapatite (HAp) particles were sterilized by autoclaving and incorporated into GelMA precursor solutions at final concentrations of 5, 10, and 15 μg/mL. To achieve uniform particle dispersion, GelMA and GelMA–HAp mixtures were sonicated at 60 °C for 30 min before use. The precursor solutions were pipetted into molds and photocrosslinked under UV light (365 nm, 80% intensity, 20 s).

Surface and cross-sectional morphologies of the hydrogels and HAp particles were examined using scanning electron microscopy (SU8600, Hitachi, Tokyo, Japan; Smart Materials Research Center for IoT supported by the Korean Basic Science Institute, NFEC-2023-02-285654). Prior to imaging, hydrogel samples were freeze-dried and sputter-coated with platinum. Chemical composition and functional group analysis were performed using Fourier-transform infrared spectroscopy (FTIR; Thermo Fisher Scientific iS50) with an ATR accessory. Hydrogel samples were freeze-dried and finely powdered prior to measurement.

### 4.3. Mechanical Analysis of Hydrogels

The viscoelastic properties of GelMA and GelMA–HAp hydrogels were measured using a rheometer (MCR92, Anton Paar, Graz, Austria) at 37 °C. Frequency sweep tests were conducted at 1% strain over a frequency range of 0.1 to 10 Hz to obtain storage modulus (G′) and loss modulus (G″). Amplitude sweep tests were performed at 1 Hz with shear strain ranging from 0.1% to 10% to assess the linear viscoelastic region.

Compressive mechanical properties were assessed using a Universal Testing Machine (UTM; Instron, Norwood, MA, USA). Cylindrical hydrogels (10 mm diameter, 8 mm height) were compressed at a rate of 1 mm/min up to 40% strain. All samples were compressed to the same strain limit without fracture or observable structural failure. Compressive modulus was calculated from the linear regions of stress–strain curves at 10–15% and 30–35% strain.

### 4.4. Spheroid Formation, Hydrogel Encapsulation, and Cell Viability Assessment

SAOS-2 osteoblast-like cells were cultured in DMEM supplemented with 10% FBS and 1% P/S at 37 °C in a humidified atmosphere containing 5% CO_2_. Cells at passage 6 were used for all experiments. For spheroid formation, agarose molds were prepared by dissolving 1 g agarose in 5 mL deionized water and heating until fully melted. SAOS-2 cells were detached using trypsin-EDTA, centrifuged, counted, and seeded at 1 × 10^5^ cells per well (75 µL per mold well). After a 30 min aggregation period, 1 mL DMEM was gently added, and spheroids were formed over 4 days with medium changes every 2 days.

Formed spheroids were collected by gentle pipetting, allowed to settle, and resuspended in GelMA or GelMA–HAp precursor solutions. A 20 µL aliquot of spheroid-containing hydrogel was placed in a 48-well plate and photocrosslinked under UV light (365 nm, 80% intensity, 20 s). Following encapsulation, osteogenic medium (DMEM supplemented with 10% FBS, 1% P/S, 10 mM β-glycerophosphate, 50 μg/mL ascorbic acid, and 100 nM dexamethasone) was added and replaced every 2 days.

Cell viability and proliferation were quantified using the CCK-8 assay at Days 1, 4, and 7. Hydrogels were washed with DPBS, incubated with 10% CCK-8 solution in DMEM at 37 °C for 2 h, and absorbance of 100 µL supernatant was measured at 450 nm using a microplate reader. For Live/Dead staining, hydrogels were immersed in staining solution containing Calcein AM (20 µL) and Ethidium Homodimer-1 (5 µL) diluted in 15 mL DPBS. After 2 h incubation, samples were washed and imaged using a fluorescence microscope (EVOS M5000, Thermo Fisher Scientific).

Statistical analysis was performed using GraphPad Prism 10. Data are presented as mean ± SD from three independent experiments (*n* = 3). Statistical significance was determined by one-way ANOVA followed by Tukey’s post hoc test, with *p* < 0.05 considered significant.

### 4.5. Osteogenic Differentiation Assays (ALP and ARS)

Early osteogenic differentiation was assessed by measuring alkaline phosphatase (ALP) activity at Days 7 and 14. Hydrogels were washed with DPBS, lysed with RIPA buffer, and centrifuged to collect supernatant. ALP activity was measured using an ALP assay kit according to manufacturer instructions. Samples were incubated with ALP substrate buffer at 37 °C for 2 h, and absorbance was recorded at 405 nm using a microplate reader.

Late-stage mineralization was evaluated using Alizarin Red S (ARS) staining at Day 14. A 2% ARS solution was prepared, sonicated, filtered, and adjusted to pH 4.2. Hydrogels were washed with DPBS, fixed in 4% paraformaldehyde for 30 min, and rinsed with deionized water. Samples were stained with ARS solution for 5 min at room temperature, and excess dye was removed with repeated deionized water washes. Mineral deposition was assessed qualitatively under bright-field microscopy (EVOS M5000, Thermo Fisher Scientific).

## Data Availability

The data generated or analyzed during this study are available from the corresponding author on reasonable request.
